# Cardiorenal Interorgan Assessment via a Novel Clustering Method Using Dynamic Time Warping on Electrocardiogram: Model Development and Validation Study

**DOI:** 10.2196/73353

**Published:** 2025-08-12

**Authors:** Sally Zhao, Zhan Ye, Bhavna Adhin, Matti Vuori, Jari Laukkanen, FinnGen, Sudeshna Fisch

**Affiliations:** 1 Pfizer (United States), New York, United States; 2 Pfizer (United States), 1 Portland St, Boston, MA, United States, 1 6175513000; 3 Pfizer (United States), Austin, TX, United States; 4 Turku University Hospital, Turku, Finland; 5 Institute for Molecular Medicine Finland, Helsinki, Finland; 6 Institute of Clinical Medicine, University of Eastern Finland, Kuopio, Finland; 7 Department of Medicine, Wellbeing Services County of Central Finland, Jyväskylä, Finland; 8 University of Helsinki, Helsinki, Finland

**Keywords:** data analysis, clustering, electrocardiogram, heart failure, chronic renal insufficiency, ECG, cardiorenal

## Abstract

**Background:**

The heart and kidneys have vital functions in the human body that reciprocally influence each other physiologically. Pathological changes in 1 organ can damage the other. Epidemiologic studies show that greater than 50% of patients with heart failure (HF) have preserved ejection fraction (HFpEF). Additionally, 1 in 6 patients identified as having chronic kidney disease (CKD) also has HF. Thus, it is important to be able to predict and identify the cardiorenal relationship between HFpEF and CKD.

**Objective:**

Creating an electrocardiogram (ECG)-enabled model that stratifies suspected patients with HFpEF would help identify CKD-enriched HFpEF clusters and phenogroups. Simultaneously, a minimal set of significant ECG features derived from the stratification model would aid precision medicine and practical diagnoses due to being more accessible and widely readable than a large set of clinical inputs. Furthermore, the validation of the existing cardiorenal relationship using this ECG-enabled model may lead to better biological understanding.

**Methods:**

Using unsupervised clustering on all extractable ECG features from FinnGen, patients with an indication of HFpEF (filtered by left ventricular ejection fraction [LVEF] values ≥50% and N-terminal pro B-type natriuretic peptide [NT-proBNP] values >450 pg/mL) were categorized into different phenogroups and analyzed for CKD risk. After isolating significant predictive ECG features, unsupervised clustering and risk analysis were performed again to demonstrate the efficacy of using a minimal set of features for phenogrouping. These clusters were then compared to clusters formed using dynamic time warping (DTW) on raw ECG time series electrical signals. Afterward, these clusters were analyzed for CKD enrichment.

**Results:**

The PR interval and QRS duration stood out as significant features and were used as the minimal feature set. After generating and comparing clusters (k-means with all extracted ECG features, k-means with a minimal feature set, and DTW with full lead II ECG waveform), the DTW-generated clusters were most stable. ANOVA analysis also showed that several HFpEF clusters exhibited a deviation of CKD risk from baseline, allowing for further trajectory analysis. Specifically, the creatinine levels (a proxy for CKD) of several DTW-created clusters showed significant differences from the average. Based on the Jaccard score, the DTW clusters also showed the greatest alignment to baseline comparison clusters created by clustering on creatinine. In comparison, the other 2 sets of clusters (created by all extracted ECG features and the minimal set) performed similarly.

**Conclusions:**

This project validates both the known cardiorenal relationship between HFpEF and CKD and the importance of the PR interval and QRS duration. After exploring the use of ECG data for patient clustering and stratification, DTW clustering with lead II waveforms resulted in the most clinically meaningful clusters in the context of HFpEF and CKD. This methodology may prove useful in exploring ECG clustering applications outside of HFpEF as well.

## Introduction

### Background

This paper examines the relationship between a specific subtype of heart failure (HF), heart failure with preserved ejection fraction (HFpEF), and chronic kidney disease (CKD) marked by progressive renal failure [1-3], emphasizing the pathophysiological connections. It explores the potential use of technology in trials and clinical management by leveraging simple and widely accessible clinical tools such as electrocardiograms (ECGs) to predict the underlying risk in subgroups and to enable earlier and more precise interventions. Such interventions can improve patient outcomes for chronic diseases of cardiovascular or overlapping renal origin [4-6].

### Biological Rationale

**Figure 1.**
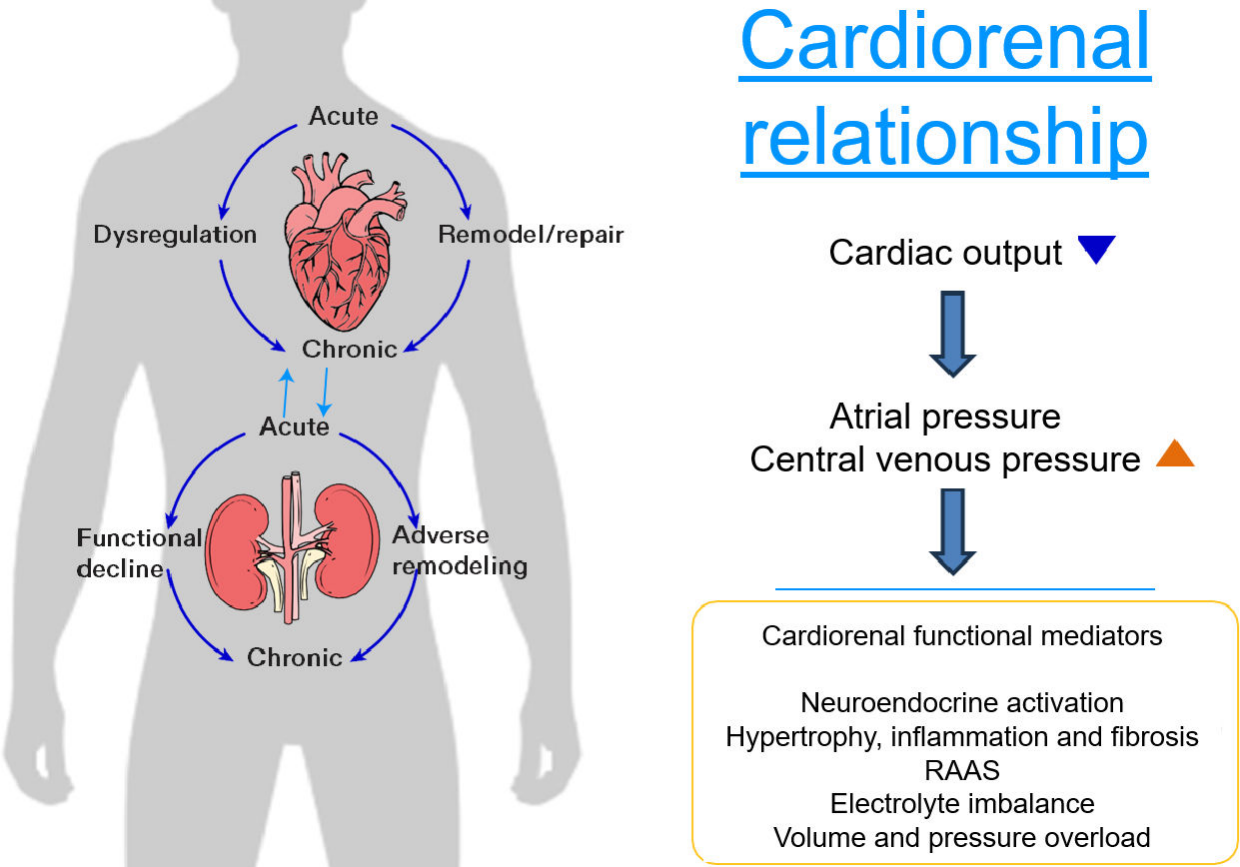
Illustration of the cardiorenal relationship and functional mediators.

HF and renal failure are 2 interrelated conditions that often coexist, significantly impacting patient outcomes [7-9]. The intricate and bidirectional relationship between the heart and kidneys illustrated in Figure 1, often referred to as the cardiorenal axis, underscores the significant unmet need to understand how dysfunction in 1 organ can precipitate or exacerbate dysfunction in the other, so that patients at risk for progression can be precisely diagnosed for earlier intervention before full onset of disease, thus improving the odds of better patient outcome [10].

HF, which is marked by the heart’s inability to pump blood effectively, initiates a series of physiological changes that can negatively impact renal function. Reduced cardiac output in HF leads to decreased renal perfusion, triggering compensatory mechanisms such as the activation of the renin-angiotensin-aldosterone system and sympathetic nervous system [11,12].

Although these mechanisms initially aim to preserve renal function and maintain blood pressure, their chronic activation can produce adverse effects, including increased sodium and water retention, further worsening heart failure symptoms and contributing to renal impairment [13,14]. HFpEF is characterized by the heart’s inability to relax (diastolic defect) and fill properly, leading to heart failure symptoms despite a normal ejection fraction (systolic function).

Conversely, renal failure can significantly affect cardiac function. The kidneys regulate fluid and electrolyte balance, blood pressure, and the elimination of metabolic waste products. When renal function declines, fluid overload, electrolyte imbalances, and the accumulation of uremic toxins can occur, adding strain on the heart. This may lead to worsening HF, creating a cycle of deteriorating cardiac and renal function. This bidirectional nature of the heart-kidney interaction underscores the complexity of managing patients with both HF and renal failure. The ability to better predict risk in these vulnerable cohorts would be beneficial for disease understanding and clinical trial recruitment.

### ECG for Clustering

Recent advancements in artificial intelligence and machine learning have shown potential in predicting and managing the risks associated with cardiorenal syndrome. Artificial intelligence–enabled models, for example, using ECG and biomarkers, have demonstrated promise in clinical care settings [15,16].

An additional use of such an ECG model in drug development could be to identify patients with HFpEF who may demonstrate a potential risk profile for worsening kidney function based on a screening ECG. Such patients may demonstrate other comorbidities such as hypertension, diabetes, or obesity, as stated earlier. This underlying and hidden risk can be revealed early for an improved understanding of the associated risks with renally cleared cardiovascular drugs. The cardiac-specific electric signals provide significant discriminatory power for endophenotyping within the broader HFpEF spectrum [17]. By leveraging machine learning-based unsupervised cluster analysis, this study aims to phenomap patients with HFpEF, enhancing the understanding and prediction of cardiorenal risk through a generalizable and interpretable ECG-based machine learning model.

Evaluating the importance of ECG features for unsupervised clustering can be difficult. A lot of tabular health care data, including the extracted ECG features, are highly correlated, which may lead to poor clustering results in practice. This also makes feature selection through Lasso very difficult. Reducing dimensionality often makes interpretability difficult as well, which is especially an issue for health care research. Thus, finding an unsupervised method that clusters high-dimensional correlated data through feature selection rather than dimension reduction would be very helpful [18].

### Dynamic Time Warping With ECG

Dynamic time warping (DTW) measures the similarity between 2 temporal sequences and, thus, can be used as a metric for clustering temporal sequences. By creating a nonlinear alignment of time sequences that are of different lengths or exhibit time shift, it can calculate the Euclidean distance between points of the 2 warped sequences. DTW has been frequently used for longitudinal and trajectory analysis, including in health care settings [19]. Due to the temporal nature of ECGs, DTW has been used to classify ECG frames and has been shown to be effective in finding nonlinear clusters of ECGs [20]. Previous literature and studies for DTW ECG clustering use the lead II recording wavelengths due to the lead II wavelength being generally considered the best view of the electrical signals because of the electrode’s placement [19].

ECGs are particularly appropriate for this combination of DTW-supported unsupervised clustering and feature analysis. First, the assumption that patients themselves, rather than the extracted ECG values, are independent and identically distributed is much easier to meet. This may make unsupervised clustering results more promising than on extracted ECG data (which would be correlated tabular data). By treating each ECG record as a singular data point, we can perform DTW on the ECG as a time series. DTW is a technique that calculates the optimal match between given sequences (it can be thought of as clustering curves via distance rather than individual data points). This way, different clusters of ECG records can be formed and then analyzed for disease endpoint incidence, cluster stability, and other performance metrics.

Many studies focus on the relationship between HFpEF and electrocardiographic (ECG) features, but our work attempts to explore the cross-organ interactions between the heart and kidney in HFpEF by using an algorithm that uses specific features within ECG features as novel predictors of cardiorenal risk. Small changes in the electroconductance system of the heart can have a big impact on the hemodynamic load on the heart, exacerbating the load, and over time, affecting volume overload [10] on the kidneys. However, such small changes in ECG, which may appear early, may not always be clinically apparent and can often be missed. In patients with HFpEF, clinical phenotype can be associated with diabetes, hypertension, and obesity [21]. Using an ECG model to identify nonobvious patterns in the electrical signals of the heart captured through a standard ECG (12 leads) can serve as indicators for left ventricular dysfunction associated with HFpEF and can potentially also identify potential signals for renal risk in a subset of patients. This study seeks to validate the established cardiorenal relationship through ECG data analysis and investigate a novel approach for clustering in HFpEF subgroups using ECG data.

## Methods

### Ethical Considerations

The real-world electronic health data used from the FinnGen database, provided for analysis, were already anonymized by FinnGen. The ethics status, data collection process, consent process, and approvals can be seen in the original study [22]. This study’s data analysis was approved by the FinnGen Committee. Due to this, there was no need to seek further ethics board approval.

### Dataset Description

The FinnGen study is a large-scale genomics initiative that has analyzed over 500,000 Finnish biobank samples and correlated genetic variation with health data to understand disease mechanisms and predispositions. The project is a collaboration between research organizations and biobanks within Finland and international industry partners [22]. This public-private partnership aggregates data from 9 different Finnish biobanks, research institutes, university hospitals, and 13 international pharmaceutical partners along with the Finnish Biobank Cooperative. Sample collection and data releases began in 2017, and the main phase of sample collection ended with FinnGen2 in 2023.

The Expansion Area 3 (EA3) studies aim to collect data on diseases that may not be present within existing FinnGen registries. Among these data is the EA3 Heart Failure Study cohort, which compiles relevant cardiovascular data from 4‐6 participating hospital biobanks. The study contains ECG files, ejection fraction values (40,809 individuals), and laboratory measurements (40,024 individuals) with B-type natriuretic peptide (BNP), proBNP, and creatinine values. Since the study is aggregated from several different existing biobanks, not all individuals have the same laboratory measurements. This project used the EA3 Heart Failure study, individuals with ECG, ejection fraction, and laboratories with creatinine and proBNP values all present.

**Figure 2.**
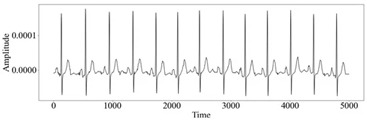
A sample lead II ECG wave. ECG: electrocardiogram.

The methods for extracting relevant features are changing and evolving over time [23], but the idea remains the same. The ECG follows a characteristic PQRST wave that occurs in a periodic pattern. Each peak, trough, or section of this wave represents electrical signals of the heart and can be evaluated for further meaning. Clinicians can look at the ECG visually for meaning, but signal processing techniques can also retrieve the mathematical values from the waves themselves. Since there are multiple electrodes for measuring the ECG, this is usually taken from lead II signals (Figure 2) [24] or aggregated.

### Data Preprocessing

**Figure 3.**
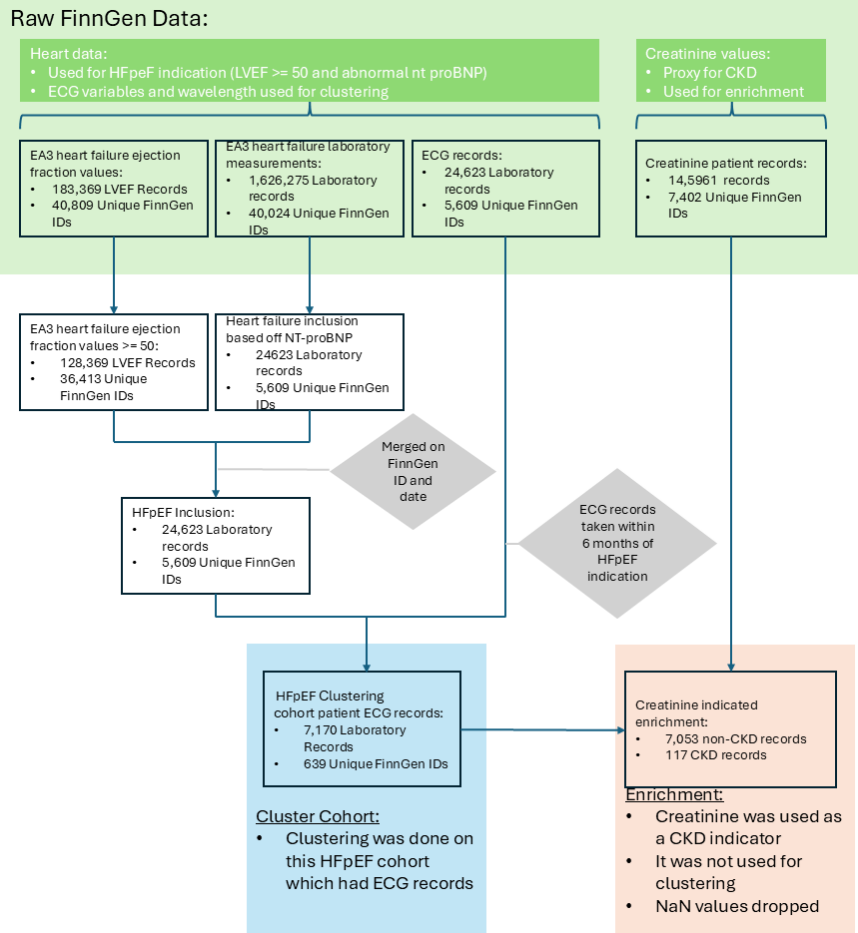
An illustration of the data processing flow and the sources of the dataset. CKD: chronic kidney disease; ECG: electrocardiogram; Expansion Area 3; HFpEF: heart failure with preserved ejection fraction.

**Figure 4.**
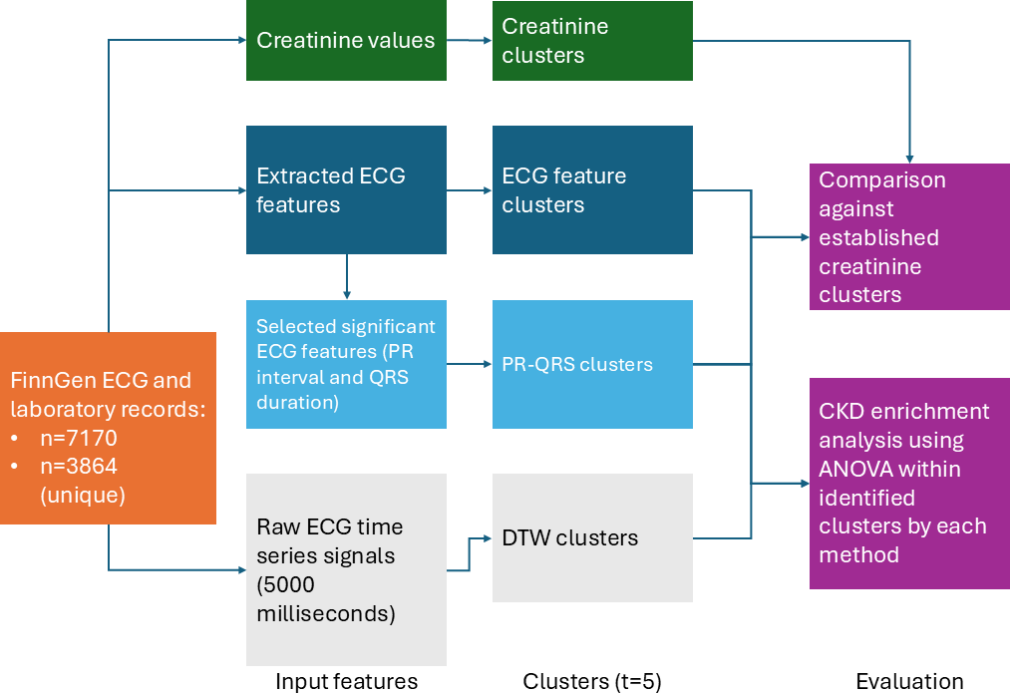
An illustration of the methodology workflow. FinnGen ECG and laboratory records are used to create different sets of clusters: Creatinine clusters (hierarchical clusters based off creatinine labs to be used as a baseline indication for CKD), ECG feature clusters (clusters created using all extracted ECG features), PR-QRS clusters (clusters created using top variables determined to be significant in predicting CKD in HFpEF), and DTW clusters (clusters created using DTW on the raw ECG time series). For each set of clusters, 5 groups were formed. CKD: chronic kidney disease; DTW: dynamic time warping; ECG: electrocardiogram; HFpEF: heart failure with preserved ejection fraction.

The dataset was restricted to the population with HFpEF (Table 1). This was determined by selecting individuals with ejection fraction values greater than or equal to 50 and exhibiting HF at that time (Figure 3). All selected individuals were verified to have HF diagnoses (either I9 HF or I9 HF NS). Only ECGs recorded within a 6-month period of the heart failure diagnosis or laboratory indicative of HF were used in this study. Creatinine values were used as a proxy for the presence of CKD. The EA3 HF Cohort contained 1,626,275 laboratory records for 40,024 unique individuals. Among these, there were 7170 complete ECGs (extracted ECG values and raw ECG signals) and creatinine laboratory records for 3864 unique individuals with HFpEF (Figure 3). The raw lead II ECG signals each contained 5000 points of data and were taken from individual ECG files within the EA3 HF cohort. These signals were directly input into the DTW algorithm. It was these ECG and laboratory records that were used for clustering and analysis (Figure 4). Specifically, the data groups to be clustered included extracted ECG values, a minimal set of extracted ECG values, and raw lead II signals.

Clusters created through hierarchical clustering on creatinine served as a baseline for CKD risk, against which the ECG-enabled clusters were compared. This comparison aimed to evaluate the effectiveness of ECG-enabled clusters in stratifying patients with CKD risk within the context of HFpEF.

**Table 1.** A characteristics average table to give a better understanding of the clustering cohort. This cohort is comprised of individuals who have characteristics that indicate HFpEF (LVEF≥50% and NT-proBNP>450 pg/mL).

Clustering cohort baseline characteristics	Values
Age (years), mean (SD)	71.16 (11.32)
Women, n (%)	3478 (48.5)
BMI (kg/m^2^), %	28.4
NT-proBNP (pg/mL)	2018.1
LVEF^a^ (%)	60.25
Creatinine (µmol/L)	103.02
Height (cm)	170.3
Weight (kg)	82.7
Current smoker, n (%)	882 (12.3)
Chronic kidney disease, n (%)	115 (1.6)

a LVEF: left ventricular ejection fraction.

b NT-proBNP: N-terminal pro B-type natriuretic peptide.

### Statistical Analysis

**Figure 5.**
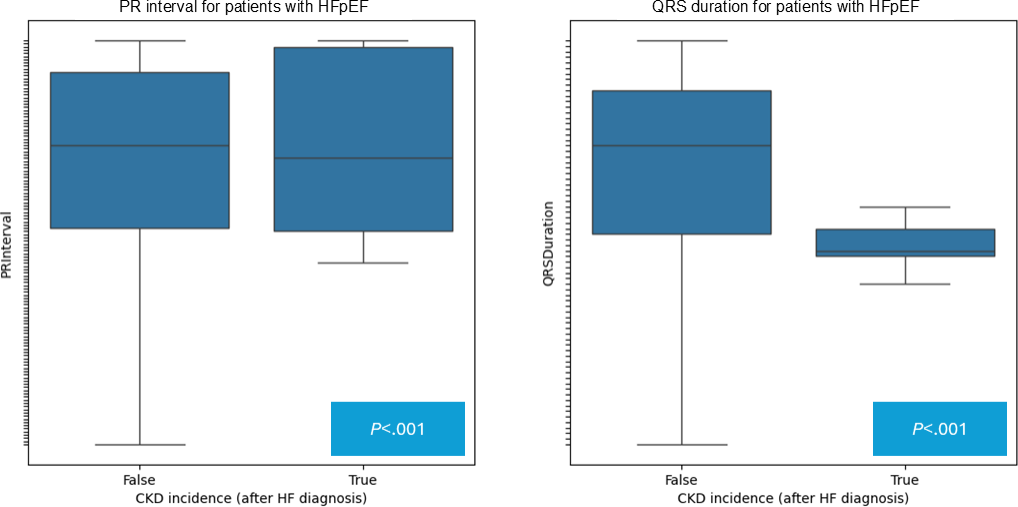
Distribution of significant extracted ECG values (QRS Duration and PR Interval) between patients who are CKD-positive and CKD-negative. CKD: chronic kidney disease; ECG: electrocardiogram; HF: heart failure; HFpEF: heart failure with preserved ejection fraction.

Univariate analysis was first performed for exploratory purposes. Distributions and averages of the extracted ECG values (ie, ventricular rate, PR interval, QRS duration, QT corrected, P axis, R axis, T axis, QRS count, Q onset, Q offset, P onset, P offset, T offset, atrial rate, and QT interval) were calculated. A 1-tailed *t* test was conducted for all extracted ECG variables to determine if there was a significant difference between patients with non-CKD and CKD HFpEF ECG values. It was shown that all extracted features were indeed significantly different with a significance level of .05 (Figure 5). Survival analysis was also conducted using a Cox proportional hazard model and a Kaplan-Meier curve.

**Figure 6.**
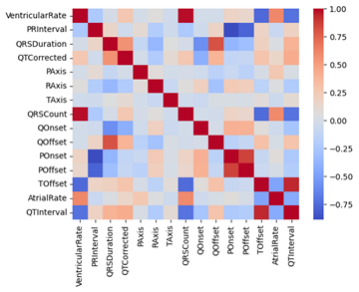
A heatmap showing the correlation between extracted ECG features. ECG: electrocardiogram.

Multivariate analysis of the extracted features was also conducted. It must be noted that the method may not be wholly accurate because the correlation between the extracted features is derived from the ECG waveform itself and can have some overlap (Figure 6). It must be noted that the method may not be wholly accurate because of the correlation between the extracted features. Additionally, these features lose some information in terms of the time component. However, it provides good exploratory insight into which combination of features would perform well for both CKD prediction and patient phenotyping.

### Determining the Number of Clusters

**Figure 7.**
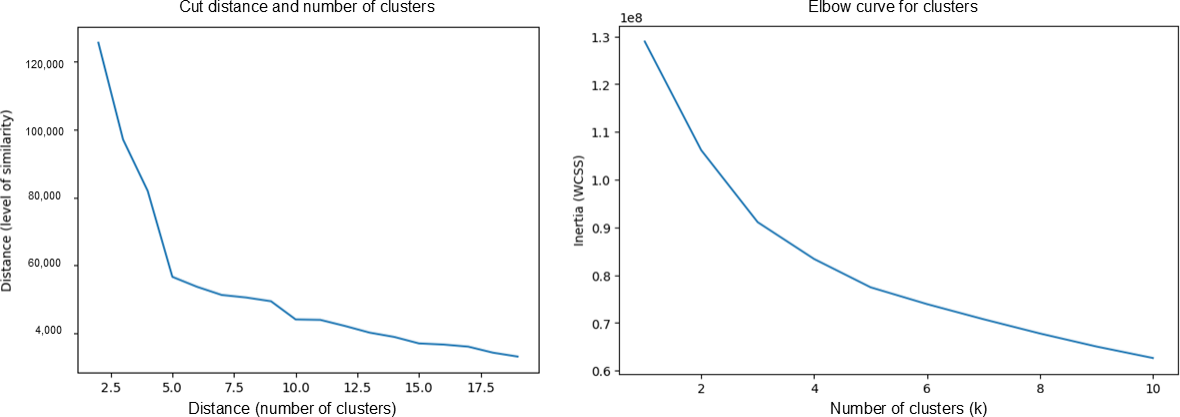
Elbow curves for extracted feature and DTW hierarchical clustering. This was used to determine the optimal number of clusters (t=5). The number of clusters was determined using these elbow charts and by seeing where the reduction in inertia or cut threshold has diminishing returns. DTW: dynamic time warping. WCSS: within-cluster sum of squares.

To determine the number of clusters created, elbow curves (Figure 7) were created and analyzed to determine which number of clusters would be optimal. As both elbow charts exhibit diminishing returns at around the 5-cluster mark, it was determined that 5 clusters would be generated and compared.

### Extracted Feature Clustering

Several methods were used, including principal component analysis (PCA), significance, and Lasso feature selection. Relevant hyperparameters (eg, α for Lasso dropout) were tuned iteratively to find the top 5 (5 is predefined) features. For PCA, 50 simulations were run for stability and robustness purposes. In every simulation, the FinnGen data were randomly sampled (training=0.8, 80% from all data) and PCA was conducted on this random sample to select the top 5 ranked features. The top 5 features in order are: ventricular rate, PR interval, QRS duration, QT corrected, and P axis. As for Lasso regression, the top features were QRS duration, QT interval, R axis, QT corrected, and PR interval. In terms of significance, the top features were ventricular rate, PR interval, QRS duration, QT corrected, and P axis, with the average explained variance reported in Table 2 on the top features identified from multiple methods. From this exploration, we can see that the PR interval and QRS duration were always selected as top features.

**Table 2.** A table ranking component (electrocardiogram variable) importance by average explained variance. PR interval and QRS duration are among the top 3 extracted variables. Ventricular rate was not chosen because it was not selected among the top 5 features during Lasso regression.

PCA^a^ component	ECG^b^ variable	Average explained variance
0	Ventricular rate	0.276
1	PR interval	0.251
2	QRS duration	0.156
3	QT corrected	0.122
4	P axis	0.091

a PCA: principal component analysis.

b ECG: electrocardiogram.

However, despite bootstrapping for stability and generalizability, the clusters formed from PR interval and QRS duration had low stability and consensus, despite performing better than clustering on all features. This is probably due to the correlated nature of the extracted ECG features. Thus, hierarchical clustering using DTW distance calculations was also performed to consider the correlation.

### DTW Clustering

The ECG lead II data were split into a training, validation, and test sets, each with 500 different unique individuals. Every patient’s lead II data included 5000 signals. These were then clustered using hierarchical clustering with the Ward method. The Ward method was chosen because it is close in functionality to k-means, which makes it intuitive and interpretable. However, it also performs better than k-means on uncovering clusters of uneven size and irregular non-spherical clusters. Exploratory analysis has shown the clusters to be uneven and not guaranteed to be regular, which makes the Ward method a good fit.

**Figure 8.**
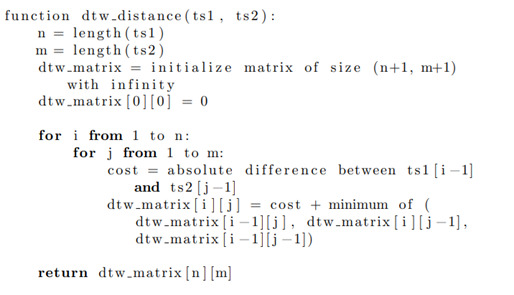
Pseudocode demonstrating the flow of the DTW algorithm and the generation of the matrix used for determining similarity between two separate time series. DTW: dynamic time warping.

DTW was used to determine the similarity between two time series. In this case, the time series would be the lead II ECG signals. This was done by calculating the Euclidean distance between each point after finding the best alignment of the 2 sequences (Figure 8). In order to find the best alignment or shift, the distance between pairs of points is compared, and the minimum distance is taken. This is done for the entirety of the 2 sequences. After creating a matrix representation for the costs associated with each alignment, the algorithm returns the minimum cost—that is, the minimum distance between aligned time series. For the regular ECGs, little shift was needed to occur. No normalization techniques were applied to the ECG lead II records. For faster processing speeds, the code was parallelized using the Python (Python Software Foundation) multiprocessing module.

Afterward, creatinine values were associated with every ECG, and the average creatinine value was calculated for every cluster. The average creatinine value of the population with HFpEF was 101.51 umol/l. Additionally, all clusters were validated through bootstrapping the data. Multiple subsets of 500 were created and clustered. This was performed to measure the stability and generalizability of the various clusters. Since the k-means and hierarchical algorithms are not consistent and exact in their results every time, this was necessary to ensure that the observed clusters (and their associated creatinine values) remained stable.

### Creatinine Clusters

Since all clusters were created in an unsupervised manner, there was no true baseline to compare the cluster labels to. Thus, the 5 clusters formed by hierarchical clustering on the creatinine laboratory values were created as a stand-in. Being a waste product from muscle and protein breakdown, creatinine levels can serve as an estimation of kidney function and a metric for CKD. Thus, comparing the extracted ECG value and DTW-formed clusters to these creatinine-based clusters can demonstrate the former’s capabilities in demonstrating creatinine enrichment and, subsequently, CKD enrichment.

Cluster stability was measured using the silhouette score [25], co-cluster occurrence (Jaccard score), and Rand index [26]. For the hierarchical clustering on the DTW-determined distance, the Jaccard index was determined to be a more relevant metric than the silhouette score. Compared to naively clustering on extracted features, the clusters were shown to be more stable.

Average features were calculated for every ECG cluster. ANOVA (Table 3) was conducted to determine any significant extracted feature difference between the clusters.

**Table 3.** ANOVA test on all extracted ECG^a^ values between the 5 clusters identified in the ECG feature clusters.

ECG variable	*F* test (*df*)	*P* value
Ventricular rate	46.945 (4, 495)	<.001
PR interval	2.466 (4, 495)	.062
QRS duration	42.311 (4, 495)	<.001
QT corrected	14.901 (4, 495)	<.001
P axis	.498 (4, 495)	.684
R axis	61.873 (4, 495)	<.001
T axis	.226 (4, 495)	.878
QRS count	47.793 (4, 495)	<.001
Q onset	15.444 (4, 495)	<.001
Q offset	28.054 (4, 495)	<.001
P onset	5.614 (4, 495)	.001
P offset	3.295 (4, 495)	.020
T offset	28.427 (4, 495)	<.001
Atrial rate	14.475 (4, 495)	<.001
QT interval	29.769 (4, 495)	<.001

a ECG: electrocardiogram.

## Results

### Clustering on Extracted ECG Features

The top 2 features determined are QRS duration and PR interval. Both of these features were unique among the top 5 ranked features in every method used (significance, Lasso, and PCA).

Although there is a difference between CKD incidence when clustering on all available ECG features compared to clustering on the significant ECG features (PR interval and QRS duration), the difference may be too small to be clinically significant.

### Clustering With DTW

**Figure 9.**
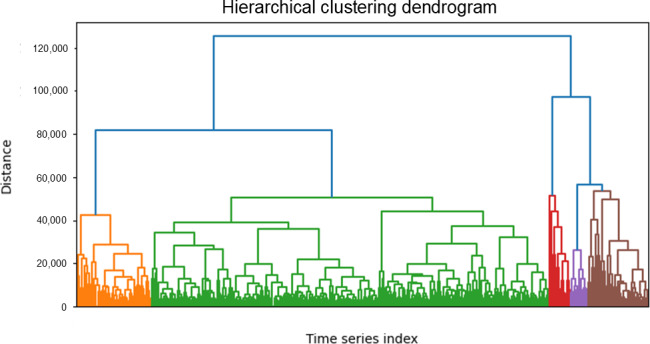
Hierarchical clustering on the validation set. It can be seen that approximately 5 clusters form which match up to the number of clusters estimated using the elbow method on the extracted ECG values. Due to the number of time series that were clustered, there were too many labels to list individually. ECG: electrocardiogram.

Regardless of how many clusters the parameter is set for with hierarchical clustering, clustering with DTW consistently yielded approximately 5 groups (Figure 9). This phenomenon could be seen in both the testing and validation sets, and also when bootstrapping.

**Figure 10.**
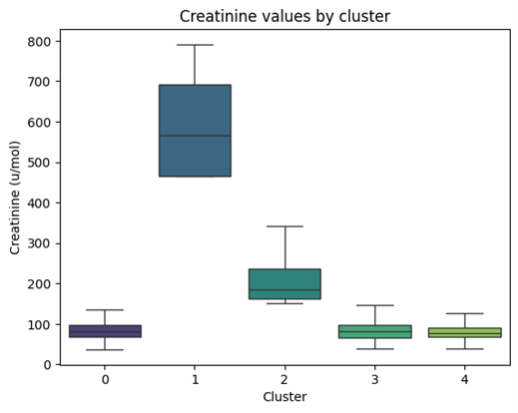
Creatinine value distribution among the 5 clusters identified in dynamic time warping clusters.

Figure 10 shows that there were creatinine differences among DTW clusters. Additionally, all extracted ECG features with the exception of P axis, T axis, and PR interval were shown to be statistically significant from cluster to cluster (Table 3). This indicates that the clusters did measurably separate the ECG time series, not just by time series shape but also by the relevant values or sections that would be extracted from the series.

**Figure 11.**
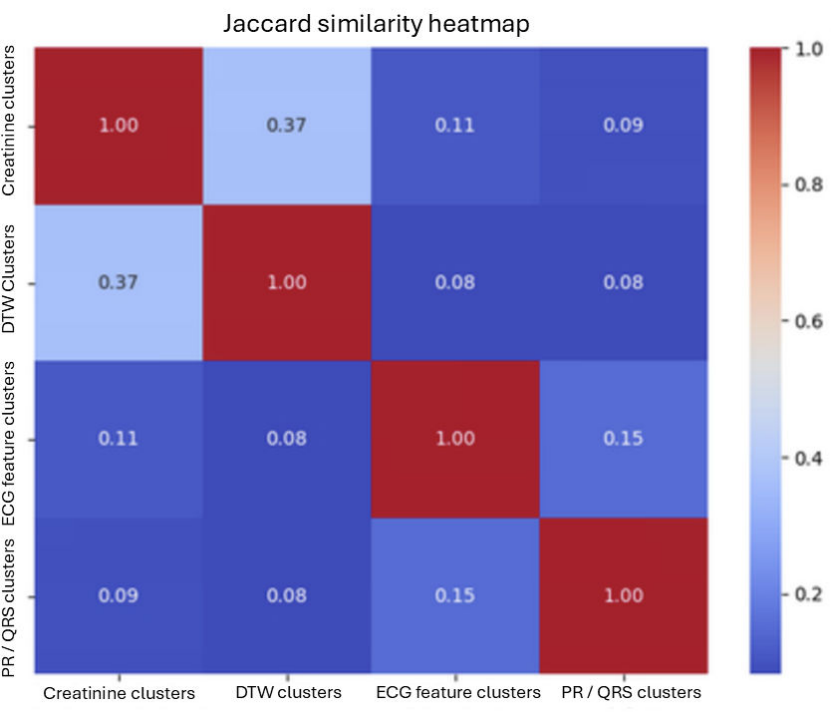
Jaccard score heatmap that compares the clusters’ similarities to each other. DTW: dynamic time warping; ECG: electrocardiogram.

Based on the Jaccard Score, the DTW-created clusters are most similar to the creatinine-based clusters (Figure 11). This indicates that it is a better stand-in for CKD enrichment than clustering solely on ECG extracted features.

## Discussion

### Principal Findings

From the results, the PR interval and QRS duration are shown to be the extracted ECG variables most associated with CKD in the HFpEF cohort. This follows the biological rationale as the PR interval and QRS complex are 2 important features of an ECG signal that capture both time and spatial dynamics of a cardiac cycle. Each aspect of the selected ECG features can provide valuable information about the heart’s function. Since the heart pumps blood systemically and into the lungs and kidneys, the electric signals may also inform about any altered hemodynamics in the latter organs [27].

The PR interval represents the time it takes for the electrical impulse to travel from the sinoatrial node, through the atria, to the ventricles. A prolonged PR interval may indicate a delay in the conduction of the electrical impulse, which can be caused by various conditions and is often suggestive of atrial abnormalities (eg, atrial volume and strain). The atrial changes can, in turn, affect subsequent changes in the ventricular contraction phase [28].

The QRS complex represents the depolarization of the ventricles, which triggers the contraction of the ventricles and the pumping of blood out of the heart into systemic circulation. The duration of the QRS complex is an important indicator of the heart’s function, and a prolongation may indicate a delay in the conduction of the electrical impulse through the ventricles, which can be caused by various conditions, including bundle branch blocks, ventricular hypertrophy, or myocardial infarction [29,30]. There is evidence to suggest that abnormalities in the PR interval and QRS duration may be associated with an increased risk of kidney disease in patients with HFpEF [28].

To make the connection from the heart to the kidney as shown in Figure 1, it is important to note that these 2 time intervals reflect the heart’s electrical conductance system and are intricately linked to cardiac relaxation, also known as diastole. During diastole, the heart fills with blood after the contraction of the ventricles, and this relaxation phase is important for maintaining adequate blood flow to the whole body, a capacity that is often lost in some patients with HFpEF.

ANOVA analysis shows significant differences between the creatinine levels of the 5 clusters generated by DTW. Not all the clusters’ creatinine levels differ significantly, but clusters 1 and 2 (Figure 10) do exhibit statistically significant differences. This means that DTW-enabled clustering was capable of stratifying patients with HFpEF with differing creatinine levels and thus differing CKD risk levels.

Despite the supported relationship between PR interval and QRS duration with CKD in HFpEF, the PR-QRS clusters did not demonstrate much similarity with the established creatinine clusters. Additionally, these PR-QRS clusters demonstrated low stability and less pronounced CKD incidence rates among the individual clusters. However, the PR-QRS clusters’ Jaccard similarity with the creatinine scores (0.09) and silhouette scores was similar to that of the ECG Feature clusters’ Jaccard similarity (0.11) with the creatinine scores and silhouette scores. This indicates that clustering on PR-QRS performs similarly to clustering on all extracted ECG features—further demonstrating that a relationship exists between PR interval and QRS duration with CKD in HFpEF. Nevertheless, due to the cluster instability and low Jaccard similarity with the creatinine baseline, it could not be used to inform clinical decisions.

DTW clusters performed much better than the PR-QRS clusters and ECG feature clusters in terms of Jaccard similarity (0.37) to the established creatinine baseline. Although the cluster groupings were not perfectly equivalent, the similarity demonstrates the potential application of using DTW for ECG clustering. This is intuitively expected due to the DTW being able to fully use the entire ECG signal. Previous literature has also supported the clustering capabilities of DTW for ECG and other signal-based records.

However, the specific reason why DTW performed better remains to be seen. Knowing the difference between the information DTW clustering captures compared to the information extracted ECG features clustering captures would be helpful. This could be used for better understanding of the statistical aspects of DTW application for clustering, as well as the cardiorenal relationship between HFpEF and CKD in this case. DTW’s better performance implies that the full lead II waveform contains more data than the extracted ECG features—minimal set or otherwise. This means that solely using extracted ECG features when working with ECG data analysis may lead to some loss of context.

### Limitations

There were certain limitations to the dataset, most notably with regard to its demographics. As the FinnGen study aggregates data from Finnish biobanks, the population is overwhelmingly Finnish and lacks heterogeneity that may pose a challenge to generalizability to other populations. Certain patients were also overrepresented in the dataset. The average patient with HFpEF had 5 ECGs spaced over a 1-year period. However, there were patients who had many ECG records (greater than 5) and patients with only a few ECG records. The FinnGen EA3 Heart Failure cohort is also not representative of all HF or patients with HFpEF. This means that extrapolating this project’s results to the wider population may not yield good results. There are disease differences between FinnGen and the external population—the renal and cardiovascular outcomes may not perform well.

Additionally, although all data were sourced from the FinnGen EA3 Heart Failure cohort, the cohort itself is comprised of several different biobanks, and each biobank provides differing amounts of data. ECG data were only available from the Central Finland Biobank. This meant that, while some patients had characteristics that indicated HFpEF, they could not be included in this study since they lacked ECG data. This data limitation introduces bias as it means all individual data is effectively from the Central Finland Biobank.

HFpEF itself is also difficult to diagnose, and the current diagnosis is multifaceted, with many clinical parameters. Due to a lack of diagnosis codes in the FinnGen EA3 Heart Failure cohort, clinical diagnosis of HFpEF could not be obtained. We used the FinnGen R12 cohort to confirm that the selected dataset had HF diagnostic codes (either I9 Heart Failure or I9 Heart Failure NS). Thus, using this in combination with the LVEF and NT-proBNP values (some of the main parameters for HFpEF), the selected individuals are indicated for HFpEF. Although this selected cohort may not be purely composed of clinically diagnosed patients with HFpEF, there could have also been a greater likelihood of finding an enrichment for patients with HFpEF. Furthermore, as the biological rationale behind the hypothesis is on the cardiorenal associations between the heart and kidneys, it is the specific heart features (ie, hypercontractility and failure) that are most relevant rather than a clinical diagnosis. The relationship between the heart and kidneys is already known to exist, so this cohort serves as a sort of positive control.

Due to the lack of diagnosis codes in the EA3 Heart Failure Cohort, CKD was also difficult to label. Even with diagnosis codes, it is entirely possible that this subset may be underdiagnosed. Creatinine laboratory records provided in the EA3 Heart Failure Cohort were used as proxies for CKD. Estimated glomerular filtration rate could have also been used, but these data were not as complete as the creatinine records and would have severely limited the cohort.

There are also confounding factors, such as medications or co-morbidities, that may affect both ECG readings and creatinine levels. Unfortunately, the lack of medications and co-morbidities from the dataset is a limitation that can be rectified in the future through validation on an external dataset such as the FinnGen R12 Cohort.

In addition, DTW also has its own limitations. Although it does consider the entire ECG signal, it reduces it down to a singular similarity metric (ie, distance) that may be reductive. Additionally, it is very computationally intensive and sensitive to noise. Extracted ECG values are not as sensitive to noise.

The main challenge with DTW was the time needed for calculations—it took a long time for every calculation. Since the distance needed to be calculated for every possible time series pair (of which there were 14,000 due to an average of 5 ECGs per patient), this was very time-consuming. Since all analysis took place within the FinnGen sandbox, which is a closed environment, not all packages were available. This included the conventional packages for DTW calculations. This was further parallelized using Python’s multiprocessing library for efficiency’s sake.

### Future Directions

Applying ECG clustering with DTW to other disease areas for similar cluster enrichment analysis may be fruitful. Previous literature has demonstrated the capabilities of ECG for risk prediction in both cardiovascular and non-cardiovascular disease areas. Analyzing the differences in cluster enrichment analysis with DTW and classic supervised risk prediction approaches may be informative.

Further work could also segment the ECG time signals. In doing so, each area could be examined for its contribution to the DTW-determined signal similarity or distance. This way, the parts of the ECG that most affect the DTW distance could be identified and determined. However, this method would be very computationally intensive—even more so than the current approach, which requires calculations for every pairwise ECG combination. Additionally, the FinnGen sandbox that houses the EA3 Heart Failure study only allows select Python packages to be installed and used (eg, the most popular and well-known ones such as *pandas*, *numpy*, *matplotlib*, *scikit-learn*, and more). DTW-related packages such as *fastdtw*, *dtaidistance*, or *tslearn* are not available on the server and must be manually coded and parallelized. This work would also need to be done for any signal segmentation DTW analysis.

Other future work involves incorporating DTW distance as a representation into autoencoders. Previous literature in both ECG and non-ECG areas has shown autoencoders to be accurate for disease trajectory and risk assessment. Incorporating not just the extracted ECG signals into autoencoders, but perhaps also a DTW score can be helpful.

Additionally, this study did not consider all comorbidities that are known to be associated with HFpEF [1], nor did it account for associated changes due to medication use in the selected cohort, for example, blood pressure control. As such, the study has limited its scope to cardiac-specific criteria and specific analytes (eg, creatinine in its methodology to understand the direct cardiorenal association). In the future, we may want to understand the potential synergies and constraints derived from adding more features, which may improve the accuracy of the prediction. In particular, we may want to evaluate the combinatorial effect of underlying physiology, medication, preexisting diseases, urinary and blood-based metabolic and protein biomarkers, and genetics that may interact with each other and our selected features. While lack of various associated clinical data may be a limitation of this study, we propose that our approach minimizes noncardiac effects that may often mask the true association between organs, for example, changes in blood pressure or pulmonary changes that may also be seen in HFpEF that have shown as a phenogroup in earlier studies [17], do not stand in conflict with our methodology. As we validate our study using other public datasets, for example, the UK Biobank, we would consider expanding the scope of the analysis.

As for practical adoption for clinical practice, the model itself is currently exploratory. The DTW-enabled clusters do show promise for patient stratification and exhibit statistically significant results in the context of HFpEF and CKD, but the associations may not be strong enough to be clinically relevant. However, the model does further validate the cardiorenal relationship and also shows ECG data to be particularly useful for phenogrouping. It showcases the relationship in a well-established cohort with good clinical phenotyping and ECG data. Specifically, the full lead II waveform is most beneficial for detecting CKD risk in patients with HFpEF—more so than any single or set of extracted ECG variables. Relying on extracted ECG variables may not give the full picture. With this information in mind, health care providers can be more aware during clinical practice. In a research setting, this information about ECG data extraction is important as it shows that not using the full waveform could lead to loss of data. The model built will be useful in expanding research beyond the relationship between ECG from patients with HFpEF to other interesting clinical phenotypes as well.

### Conclusions

From the results, the PR interval and QRS duration are shown to be the extracted ECG variables most associated with CKD in the HFpEF cohort. This follows the biological rationale as the PR interval and QRS complex are 2 important features of an ECG signal that capture both time and spatial dynamics of a cardiac cycle. The DTW application for ECG clustering would also be fruitful to explore.

## Abbreviations

CKD: chronic kidney disease
DTW: dynamic time warping
EA3: Expansion Area 3
ECG: electrocardiogram
HF: heart failure
HFpEF: heart failure with preserved ejection fraction
PCA: principal component analysis
